# Reconstructing tumor clonal lineage trees incorporating single-nucleotide variants, copy number alterations and structural variations

**DOI:** 10.1093/bioinformatics/btac253

**Published:** 2022-06-27

**Authors:** Xuecong Fu, Haoyun Lei, Yifeng Tao, Russell Schwartz

**Affiliations:** Department of Biological Sciences, Carnegie Mellon University, Pittsburgh, PA 15213, USA; Computational Biology Department, Carnegie Mellon University, Pittsburgh, PA 15213, USA; Computational Biology Department, Carnegie Mellon University, Pittsburgh, PA 15213, USA; Department of Biological Sciences, Carnegie Mellon University, Pittsburgh, PA 15213, USA; Computational Biology Department, Carnegie Mellon University, Pittsburgh, PA 15213, USA

## Abstract

**Motivation:**

Cancer develops through a process of clonal evolution in which an initially healthy cell gives rise to progeny gradually differentiating through the accumulation of genetic and epigenetic mutations. These mutations can take various forms, including single-nucleotide variants (SNVs), copy number alterations (CNAs) or structural variations (SVs), with each variant type providing complementary insights into tumor evolution as well as offering distinct challenges to phylogenetic inference.

**Results:**

In this work, we develop a tumor phylogeny method, TUSV-ext, which incorporates SNVs, CNAs and SVs into a single inference framework. We demonstrate on simulated data that the method produces accurate tree inferences in the presence of all three variant types. We further demonstrate the method through application to real prostate tumor data, showing how our approach to coordinated phylogeny inference and clonal construction with all three variant types can reveal a more complicated clonal structure than is suggested by prior work, consistent with extensive polyclonal seeding or migration.

**Availability and implementation:**

https://github.com/CMUSchwartzLab/TUSV-ext.

**Supplementary information:**

Supplementary data are available at *Bioinformatics* online.

## 1 Introduction

Cancer is widely understood as an evolutionary process (Nowell, 1976), in which gradual accumulation of somatic genetic alterations occurs in parallel with selection for mutations promoting tumor growth or other aspects of disease progression. The evolutionary history is reflected in various kinds of genetic or epigenetic variations tumor cell lineages might accumulate (Davis *et al.*, 2017). The stochastic nature of the evolutionary process typically results in high genetic and epigenetic heterogeneity both between distinct tumors (inter-tumor heterogeneity) and between cell lineages in single tumors (intra-tumor heterogeneity). A vibrant field of ‘tumor phylogenetics’ (Desper *et al.*, 1999) has arisen with the goal of using such variations to reconstruct the history of single tumors in order to better understand the evolutionary landscapes of tumor cell lineages and how individual tumors navigate them.

The general idea of tumor phylogenetics has spawned numerous variants devoted to distinct kinds or combinations of genetic data or variation type (Schwartz and Schäffer, 2017). To date, most such methods have been developed to work with bulk sequencing, in which one has mixed samples of genomic data from many tumor cells and must computationally deconvolve them to infer distinct cell lineages that can be resolved into a phylogeny (Schwartz and Shackney, 2010). Methods for deconvolutional phylogenetics of bulk genomic data have been developed predominantly for inference of genetic variation from single-nucleotide variant (SNV) data (El-Kebir *et al.*, 2015; Roth *et al.*, 2014; Yuan *et al.*, 2015). It has become increasingly apparent that copy number alterations (CNAs) are of at least comparable importance to SNVs in tumor evolution, however, prompting increasing numbers of methods for CNAs (Oesper *et al.*, 2013; Zaccaria *et al.*, 2018; Zaccaria and Raphael, 2020) or combined SNV and CNA data (Deshwar *et al.*, 2015; El-Kebir *et al.*, 2016; Li and Xie, 2015; Sashittal *et al.*, 2021). More recently, it has also been shown to be possible to infer phylogenies incorporating structural variations (SVs) (Eaton *et al.*, 2018) such as large deletions or translocations, which are more complicated to resolve phylogenetically but often key events in driving tumor evolution.

Attention in the field has increasingly turned to single-cell data (Gao *et al.*, 2016; Navin *et al.*, 2011), which can greatly improve on the limited accuracy and resolution of computational deconvolution in resolving intratumor heterogeneity and clonal progression (Kuipers *et al.*, 2017). Single-cell sequencing also has substantial drawbacks, however. Single-cell tumor phylogeny methods (e.g. Satas *et al.*, 2020; Zafar *et al.*, 2019) must contend with technical artifacts, such as allelic dropouts (Wang *et al.*, 2012). Furthermore, single-cell data, particularly single-cell DNA-seq (scDNA-seq), remains costly to gather and thus all large tumor data resources are still based on bulk sequence. Some methods have been proposed to combine bulk and single-cell sequence (Lei *et al.*, 2020; Malikic *et al.*, 2019) or other heterogeneous data combinations (Fu *et al.*, 2021; Lei *et al.*, 2021), but major sequencing efforts to date have not been designed to take advantage of such methods. Furthermore, single-cell data so far has limited ability to capture SVs as well as limited resolution for CNAs. As a result, deconvolutional methods remain important particularly for studies of variations other than SNVs.

To date, there has been no method that integrates all major variant types (SNVs, SVs and CNAs) into a single clonal lineage tree inference. This is particularly problematic as these variants typically occur together and offer complementary advantages for understanding tumor evolution. SNVs are the simplest to handle phylogenetically and are often relatively numerous. CNAs are likely more important to understanding functional adaptation in cancers (Zack *et al.*, 2013) and can be a confounding factor in interpreting SNVs correctly, but they are more challenging to interpret phylogenetically, particularly in deconvolutional settings. SVs can also have profound effects on genome function and can confound interpretation of other variant types (Li *et al.*, 2020). SVs may also be particularly useful as phylogenetic markers because they are comparatively rare, but this rarity also means they are poorly suited for phylogenetic inference on their own. So far, TUSV (Eaton *et al.*, 2018) has been the only sequencing-based tumor phylogeny method capturing SVs, which it did in conjunction with CNAs. However, it used a limited model of CNAs, for example in neglecting information on allelic specific copy number changes that are important for interpreting other variants. Most importantly, it did not incorporate SNVs, thus omitting a great deal of information that may be informative about both phylogenies and functional adaptation.

In this work, we develop a next generation tool we call TUSV-ext: the first tumor phylogeny software to accommodate SNVs, CNAs and SVs within a single phylogenetic framework and resolve all three together as markers of tumor evolution. It accomplishes this by extending the TUSV integer linear programming (ILP) framework. Major improvements over the prior work include (i) allelic segmental copy numbers and joint phasing of breakpoints and SNVs; (ii) a Dollo parsimony model of both breakpoints and SNVs, in which SNVs or breakpoints can be acquired once but potentially lost or duplicated multiple times only through copy number change; and (iii) a subsampling and assignment mechanism for productively managing larger number of variants. Extensions (i) and (ii) are especially helpful for integrated inference of SNVs and CNAs due to improvements in estimation of VAF of SNVs with current tools. We demonstrate on simulated data that the method shows generally superior performance in practice in comparison to the prior art at common subtasks, in addition to accommodating a broader set of variant types than any prior methods in this space. We further apply TUSV-ext to a published prostate cancer dataset (Gundem *et al.*, 2015) where it yields results consistent with prior findings and revealing of novel insight into how these variant types are complementary in reconstructing evolution of a single tumor.

## 2 Materials and methods

**Fig. 1.**
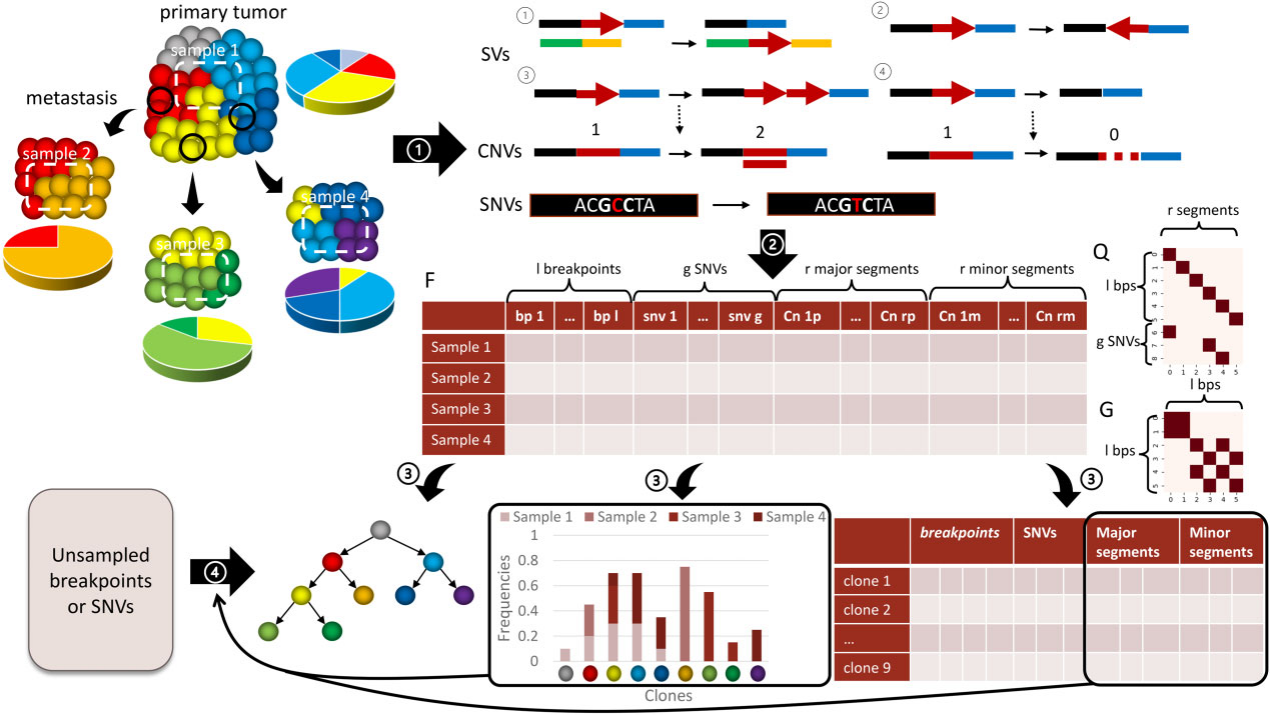
Overview of the tumor evolution reconstruction method of TUSV-ext. (1) Multi-regional samples are collected from one or more tumor sites or progressions stages, assumed to contain different compositions of a common set of clones, which we assume have been sequenced and from which various variant types have been called. (2) We subsample the structural variants and SNVs as needed and preprocess the variant information into three matrices: a variants copy number matrix *F*, a positional encoding matrix *Q*, and a breakpoints pairing matrix *G*. (3) We then apply the TUSV-ext algorithm to deconvolve the mixed samples into a set of clones each defined by a variant set, copy number profile, and frequency in each sample, as well as an inferred phylogeny on the clones. (4) By using the inferred frequencies and segmental copy number information of each clone, we assign unsampled breakpoints and SNVs to the inferred phylogeny to obtain a comprehensive evolutionary trajectory

Figure 1 provides a high-level overview of our method. Our method aims to infer genetic variation profiles (SNVs, CNAs and SVs) of multiple tumor clones and the distributions of these clones across samples from bulk DNA sequencing data. Each SV is described by a pair of breakpoints with chromosomal, positional and directional information, showing an abnormal junction compared with normal genome, which is often identified from discordant and split reads. Each CNA is described as a copy number of a genome segment, showing the level of aneuploidy of tumor sample. Each SNV is described by a location in the genome and a variant allele frequency (VAF) as estimated by the ratio of sequencing reads of alternate and reference alleles. Using the aforementioned variant types as input, we seek to assign variants to a set of inferred tumor clones and simultaneously infer a tumor phylogenetic tree representing the evolutionary history of these clones.

### 2.1 Problem statement

Our method takes as input processed variant calls with paired breakpoint ends and their estimated mean copy numbers describing SVs, allelic mean copy numbers of genome segments describing CNAs, and estimated mean copy numbers of SNVs in Variant Call Format (VCF) format. We encode these variations in three matrices, *F*, *Q* and *G*, which contain mean variant copy numbers (*F*), positional relationships between breakpoints/SNVs and copy number segments (*Q*), and pairing information for genomic breakpoints identifying which genome segments are involved in SVs for a set of genomic samples (*G*). Mathematical details of the matrices can be found in Table 1. Our goal is to deconvolve the mixed samples by minimizing an objective function:

(1) $$\min_{U,C}|F-UC|+\lambda_{1}R+\lambda_{2}S,$$

to solve for a matrix of variant copy number profiles for each clone *C* and a matrix of clonal frequencies *U* with constraints from phylogenetic trees and relationships among different variants. An L1 loss function between the reconstructed mixed profiles of variant copy numbers (*UC*) and real bulk sample(s) (*F*) defines the main term of the objective, which ensures that deconvolved clonal profiles and compositions are as similar as possible when they are mixed together under the inferred frequencies. *Q* and *G* are used in setting the constraints and but not in the objective function.

**Table 1.** Essential parameters and variables

Notation	Meaning
$F\in R_{\geq 0}^{m\times (l+g+2r)}$	$f_{p,v}$ Average copy number of variant *v* in sample *p*
$Q\in {\left\{0,1\right\}}^{(l+g)\times r}$	$q_{b,s}=1$ iff breakpoint or SNV *b* is in segment *s*, else 0
$G\in {\left\{0,1\right\}}^{l\times l}$	$g_{b_{1},b_{2}}=1$ iff breakpoint *b*_1_ and *b*_2_ are paired breakpoints deriving from the same SV
*l*	The number of breakpoints
*g*	The number of SNVs
*r*	The number of haploid copy number segments
*n*	The number of leaves in the phylogenetic tree
*N*	The number of total clones in the phylogenetic tree, N=2n−1
*m*	The number of samples
*c_max_*	The maximum allowed copy number for each segment
$C\in Z_{\geq 0}^{n\times (l+g+2r)}$	$c_{k,v}$ is the copy number of variants in clone *k*. When v∈{1,…,l}, it represents the copy number of breakpoint *v*. When v∈{l+1,…,l+g}, it represents the copy number of SNV *v*. When v∈{l+g+1,…,l+g+r} or v∈{l+g+r+1,…,l+g+2r}, it represents the copy number of segment *v* from one allele or another
$U\in R_{\geq 0}^{m\times k}$	$u_{p,k}$ is the frequency of clone *k* in sample *p*
$E\in {\left\{0,1\right\}}^{N\times N}$	$e_{i,j}=1$ iff there is a directed edge from clone *i* to clone *j* in the phylogenetic tree, else 0
$A\in {\left\{0,1\right\}}^{N\times N}$	$a_{i,j}=1$ iff clone *i* is an ancestor of clone *j* in the phylogenetic tree, else 0
$W\in {\left\{0,1\right\}}^{N\times N\times (l+g)}$	$w_{i,j,b}$ is a 0–1 binary indicator, 1 iff breakpoint or SNV *b* occur at edge from node *i* to *j* else 0
$D\in {\left\{0,1\right\}}^{(l+g)\times 2}$	*d_b_* = 1 indicates that the breakpoint *b* is in the first allele and *d_b_* = 0 indicates the second allele
$\Gamma \in Z_{\geq 0}^{N\times (l+g)\times 2}$	$\gamma_{i,b,0}$ is the segment copy number of node *i* at the position where breakpoint *b* locate in the first allele and $\gamma_{i,b,1}$ the second allele
$\Psi \in Z_{\geq 0}^{m\times (l+g)}$	$\psi_{p,b}$ is the mixed copy number of segment in sample *p* for both allele that contains the breakpoint or SNV *b*

The model uses two regularization terms, *R* and *S*, to establish penalties corresponding to an evolutionary distance measure used to favor minimum-evolution phylogenies and consistency between variant types, respectively. The term *R* is an L1 distance of the clonal copy number profiles summed over edges of the inferred phylogeny, where we seek to penalize for large evolutionary distance that would suggest an unlikely phylogeny. This term is equivalent to implicitly assuming that copy numbers evolve by gain or loss of single copies of single genome segments with uniform rate. The term *S* aims to penalize for large discrepancies between the inferred frequencies of variants and their empirically observed frequencies. The problem statement is at a high level similar to that of the original TUSV (Eaton *et al.*, 2018), although the formulation of each term is substantially extended to handle SNV markers and allele-specific copy numbers of all variants, among other changes. Due to space limits, the complete problem statement as an ILP is provided in Supplementary Methods S1. In the remainder of this section, we provide a summary of the main components of the ILP, highlighting novel extensions compared with our prior work (Eaton *et al.*, 2018).

### 2.2 Algorithm: coordinate descent

We use coordinate descent to solve for matrices *U* and *C* given input matrices *F*, *Q* and *G* as in Eaton *et al.* (2018). We first initialize the *U* matrix randomly under the constraint that $\sum_{k=1}^{N}u_{p,k}=1,\forall p\in \{1\ldots m\}$ and solve for *C* with fixed *U*. We then fix *C* and solve for *U*. These steps alternate iteratively until convergence or a maximum number of iterations are reached. Random starts are optionally allowed to reduce risk of solutions being stuck in local optima. Each step of the coordinate descent algorithm is posed in terms of an ILP optimization problem as described in more detail in the Supplementary Methods S1. Descriptions of all major parameters or variables are found in Table 1.

#### 2.2.1 Estimating clonal frequency matrix *U*

As described in Eaton *et al.* (2018), we construct an ILP describing the element-wise absolute distance between *F* and *UC*. *U* can be solved in terms of *F* and *C* by minimizing only the first term of the objective function, |F−UC|, as the two penalty terms do not depend on *U*, under the constraint that the sum of the frequencies of all clones in each sample is one (see Supplementary Method S1 Equation (S27)–(S30) for detailed ILP formulation).

To allow compatibility with other methods for purposes of comparison, we also provide a leaf-only version of the ILP where non-leaf nodes (except the root node representing the normal cell population) are constrained to have zero fractions and only the leaf nodes are allowed to have non-zero estimated frequencies.

#### 2.2.2 Estimating copy number matrix *C*

The method next fixes *U* and solves for *C* given input matrices *F*, *G* and *Q*. Because *C* encodes the variant copy number profile of each subclone, it is related to the underlying evolutionary tree structure and can be used to constrain possible trees as described in the next section.

**Phylogenetic constraints:** As in Eaton *et al.* (2018), in order to relate the copy number profiles to the phylogenetic tree *T*, we define an edge matrix $E^{N\times N}$ describing parent-child relationships in *T*, where $e_{i,j}=1$ indicates clone *i* is the direct parent of clone *j*, as well as an ancestor matrix to describe ancestor-descendent relationships in the tree, where $a_{i,j}=1$ indicates clone *i* is an ancestor of clone *j*. We assume the phylogenetic tree *T* is a binary tree without loss of generality. We impose basic phylogenetic constraints on the root, internal and leaf nodes to ensure the tree is binary (see Supplementary Method S1 Equations (S31)–(S39) for details).

Beyond the common constraints, we add new constraints to break cycles, which we enforce by requiring that for any two nodes *i* and *j*, either *i* is the ancestor of *j*, *j* is the ancestor of *i* or neither is the other’s ancestor, and that a node cannot be its own ancestor or descendent:

(2) $$a_{i,j}+a_{j,i}<=1,\forall i,j\in \{1,\ldots ,N\}$$

 

(3) $$a_{i,i}=0,\forall i\in \{1,\ldots ,N\}$$

**Subclone variant copy number constraints:** The tree describes somatic variations accumulating after descent from the root node and so we assume that the root has no breakpoints or SNVs (Equation (5)) and has a single copy of each allele in each genomic segment (Equation (6)). For algorithmic convenience, we also restrict copy numbers to a maximum value *c_max_* (Equation (4)).

(4) $$c_{k,s}\leq c_{\max},\forall k\in \{1,\ldots ,N\},s\in \{1,\ldots ,l+g+2r\}$$

 

(5) $$c_{N,b}=0,\forall b\in \{1,\ldots ,l+g\}$$

 

(6) $$c_{N,l+g+s}=1,\forall s\in \{1,\ldots ,2r\}$$

In contrast to the original TUSV, we use two temporary variables $x_{i,j,s}^{1}$ and $x_{i,j,s}^{2}$ corresponding to two original alleles, instead of one variable $x_{i,j,s}$, to define the absolute changes in copy numbers of segment *s* of two alleles on edge (*v_i_*, *v_j_*), where *e_ij_* = 1 and $x_{i,j,s}$ will define the exact absolute allelic copy number change from node *i* to *j*. In the other scenario, where *e_ij_* = 0, the temporary variables are set to be 0.

(7) $$0\leq x_{i,j,s}^{1}\leq c_{\text{max}}e_{i,j},\forall s\in \{1,\ldots ,r\}$$

 

(8) $$x_{i,j,s}^{1}\geq c_{i,s+l+g}-c_{j,s+l+g}-c_{\text{max}}(1-e_{i,j})$$

 

(9) $$x_{i,j,s}^{1}\geq -c_{i,s+l+g}+c_{j,s+l+g}-c_{\text{max}}(1-e_{i,j})$$

 

(10) $$0\leq x_{i,j,s}^{2}\leq c_{\text{max}}e_{i,j}$$

 

(11) $$x_{i,j,s}^{2}\geq c_{i,s+l+g+r}-c_{j,s+l+g+r}-c_{\text{max}}(1-e_{i,j})$$

 

(12) $$x_{i,j,s}^{2}\geq -c_{i,s+l+g+r}+c_{j,s+l+g+r}-c_{\text{max}}(1-e_{i,j})$$

We add these two copy numbers together through all segments and all edges to form a copy number evolutionary distance used in regularization term *R*:

(13) $$\rho_{i,j}=\sum_{s=1}^{r}x_{i,j,s}^{1}+\sum_{s=1}^{r}x_{i,j,s}^{2}$$

 

(14) $$R=\sum_{i=1}^{N}\sum_{j=1}^{N}\rho_{i,j}$$

**Perfect phylogeny on breakpoints and SNVs:** We constrain each pair of breakpoints so that they must appear on a common edge in the tree and impose a Dollo phylogeny constraint on SV breakpoints to ensure that each variant can only appear once as described in Eaton *et al.* (2018).

As a new feature, we add SNVs with the same Dollo assumption (Supplementary Equation (S46)). The assumption of no homoplasy is more dubious for SNVs than for SVs and may be a source of error, but one we permit out of algorithmic necessity. Therefore we define $W\in {\left\{0,1\right\}}^{N\times N\times (l+g)}$ to describe the edge on which each breakpoint or SNV occurs, where $w_{i,j,b}=1$ if variant *b* occurs at the edge from node *i* to *j* and 0 otherwise (Supplementary Equation (S44)–(S45)).

We first define a binarization operator, as described in Zaccaria *et al.* (2018) and Eaton *et al.* (2018), as follows.

$$\bar{x}=\{\begin{array}{cc} 1 & x>0\\ 0 & x=0 \end{array}$$

and then define an auxiliary variable *X* to help define *W* (see Supplementary Method S1 Equations (S42) and (S43) for detail of how the binarization operator is defined and Supplementary Equation (S44)–(S46) for Dollo phylogeny constraints on both SNVs and breakpoints).

In addition, we assume it to be very unlikely that one variant, especially a structural variant defined at base resolution, can revert so that the variant is lost autonomously. Rather, we assume losses will occur only by copy number change resulting in loss of the allele containing the variant. Therefore, we impose constraints that every loss of a breakpoint or SNV should co-occur with an allele-specific segment copy number loss for the segment in which the breakpoint or SNV lies. Similarly, we also constrain a breakpoint or SNV to be duplicated only if its corresponding genome segment is duplicated.

Allele-specific variation is a novel extension of this work and requires additional constraints. In order to assign variants to alleles, we introduce a new binary variable matrix $D\in {\left\{0,1\right\}}^{(l+g)\times 2}$ where *d_b_* = 1 indicates that the breakpoint *b* is in the first allele and *d_b_* = 0 indicates the second allele. Since the two alleles can have different copy numbers and it is important to identify common alleles in different samples, we further assume that the first segment shares a common allele in each sample on the assumption that there is unlikely to be duplication and loss simultaneously in one segment. Therefore, during preprocessing, we compare the copy numbers of all alleles and order alleles so that the larger copy number corresponds to the first allele and the smaller copy number to the second allele for all samples. We therefore define matrix $\Gamma \in Z_{\geq 0}^{N\times (l+g)\times 2}$, where $\gamma_{i,b,0}$ is the segment copy number of node *i* at the position where a breakpoint or SNV *b* is located in the first allele and $\gamma_{i,b,1}$ correspondingly for the second allele. We further constrain that for any existing breakpoint or SNV in the clone (excluding newly developed breakpoints in this branch), the breakpoint copy number change should be smaller than or equal to the corresponding copy number change at the breakpoint’s or SNV’s position.

(15) $$\gamma_{j,b,0}-\gamma_{i,b,0}\geq c_{j,b}-c_{i,b}-(2-e_{i,j}-d_{b}+w_{i,j,b})(2c_{\text{max}}+1),$$

 

(16) $$\gamma_{j,b,0}-\gamma_{i,b,0}\leq c_{j,b}-c_{i,b}+(2-e_{i,j}-d_{b}+w_{i,j,b})(2c_{\text{max}}+2)$$

 

(17) $$\gamma_{j,b,1}-\gamma_{i,b,1}\geq c_{j,b}-c_{i,b}-(1-e_{i,j}+d_{b}+w_{i,j,b})(2c_{\text{max}}+1),$$

 

(18) $$\begin{array}{c} \gamma_{j,b,1}-\gamma_{i,b,1}\leq c_{j,b}-c_{i,b}+(1-e_{i,j}+d_{b}+w_{i,j,b})(2c_{\text{max}}+2)\\ \forall i,j\in \{1,\ldots ,N\},b\in \{1,\ldots ,l+g\} \end{array}$$

**Structural variant and segment consistency:** The copy number of a breakpoint or SNV for each clone should not exceed the copy number of the segment in which it is found. However, in order to integrate allelic information, we only constrain the copy number of a breakpoint to be at most the copy number of the segment of its corresponding alleles.

(19) $$c_{k,b}\leq \gamma_{k,b,0}+(1-d_{b})c_{\text{max}}=\sum_{s=1}^{r}q_{b,s}c_{k,s+l+g}+(1-d_{b})c_{\text{max}}$$

 

(20) $$c_{k,b}\leq \gamma_{k,b,1}+d_{b}c_{\text{max}}=\sum_{s=1}^{r}q_{b,s}c_{k,s+l+g+r}+d_{b}c_{\text{max}}$$

For copy numbers of mixed samples, we define $\Psi \in Z_{\geq 0}^{m\times (l+g)}$ so that $\psi_{p,b}$ is the mixed copy number of the segment accounting for both alleles in sample *p* that contain the loci of a given breakpoint or SNV *b*.

(21) $$\psi_{p,b}=\sum_{s=1}^{r}q_{b,s}(f_{p,s+l+g}+f_{p,s+l+g+r})$$

We calculate the ratio of mixed copy numbers of a breakpoint or SNV and mixed copy number of segments as $\pi_{p,b}$ (also known as variant allele frequency VAF):

(22) $$\pi_{p,b}=\frac{f_{p,b}}{\psi_{p,b}}$$

We bias toward minimizing the distance between the estimated and actual ratios (Equation (23)) by adding a regularization term that sums the absolute differences between the two ratios for sample *p* and variant *b* (breakpoint or SNV) among all samples and variants, represented by auxiliary variable $z_{p,b}$ (Equations (24)–(26)):

(23) $$\left|\pi_{p,b}-\frac{\sum_{k=1}^{N}u_{p,k}c_{k,b}}{\sum_{k=1}^{N}u_{p,k}(\gamma_{k,b,0}+\gamma_{k,b,1})}\right|$$

 

(24) $$\Leftrightarrow z_{p,b}\geq \pi_{p,b}\sum_{k=1}^{N}u_{p,k}(\gamma_{k,b,0}+\gamma_{k,b,1})-\sum_{k=1}^{N}u_{p,k}c_{k,b}$$

 

(25) $$z_{p,b}\geq -\pi_{p,b}\sum_{k=1}^{N}u_{p,k}(\gamma_{k,b,0}+\gamma_{k,b,1})+\sum_{k=1}^{N}u_{p,k}c_{k,b},$$

 

(26) $$\begin{array}{c} \forall b\in \{1,\ldots ,l+g\}\\ S=\sum_{p=1}^{m}\sum_{b=1}^{l+g}z_{p,b} \end{array}$$

#### 2.2.3 Choice of *λ*_1_ and *λ*_2_

We set the regularization parameters $\lambda_{1}=\frac{1}{2}\frac{l+g+2r}{2r}\frac{m}{N}$ and $\lambda_{2}=\frac{1}{2}\frac{l+g+2r}{l+g}$ by simple formulas meant to approximately normalize for the expected scales of *R* and *S*. In this, we follow the original TUSV work (Eaton *et al.*, 2018), which further established that that method was not greatly sensitive to the selection of penalty parameters.

#### 2.2.4 Estimating the number of clones

Determining the number of subclones is a hard problem for which we provide an optional heuristic. The user can preset a maximum number of tree nodes and the method will infer a tree with the given maximum size then collapse nodes inferred to have zero frequency when they only have one child and collapse child nodes with zero branch length before counting the final number of uncollapsed clones. We also allow the user to predefine the subclone number directly. In practice, the method can handle clone numbers up to approximately 9 before run time becomes excessive.

### 2.3 Mapping unsampled SNVs or breakpoints to identified nodes

The full algorithm in principle provides a problem formulation that we can optimize for any dataset. However, the ILP framework can scale poorly to large numbers of variants, as may occur in highly mutable cancers. We therefore introduce a variation on the method in which we subsample the full set of variants for phylogeny inference and then map the remaining variants to the identified subclonal populations derived from the subsampled data. The intuition behind this alternative is that many variants will provide redundant information about the phylogeny and so we should not need a large number of variants for the computationally costly step of inferring the correct topology for the phylogeny. We can thus solve the phylogeny more quickly on a subset of variants and then efficiently map the remaining variants to their most likely positions on the phylogeny.

With this alternate method, we first subsample SNVs and breakpoint pairs up to a user-defined maximum of each, while keeping all CNAs. We then solve the ILP as in Section 2 to obtain an inferred tree on the variant subset, including a tree topology and copy number profile and clonal frequency of each inferred clone. We then map the remaining breakpoints and SNVs to edges of the tree given their estimated copy numbers. We do not know in which allele the remaining SNVs or breakpoints lies, but the number of possibilities is generally small. We therefore identify the most plausible edge in the tree at which each variant individually could have first been acquired by enumerating over all tree edges and choosing the one that yields the best match to the estimated copy number of that variant.

If there were no copy number changes then the VAF implied by a given edge would be half the sum of the clonal frequencies of all nodes below that edge. To account for CNAs, we consider two possibilities when a variant is acquired on the same edge as a CNA: (i) the variant was acquired before the copy number change, which results in the actual copy number of the variant being equal to the copy number of its corresponding segment. (ii) the variant is acquired after the copy number change, which results in that the actual copy number of the variant in that clone being one. To account for further copy number changes after the acquisition of a given variant, we estimate the copy number of the variant to be the expectation over possible copy numbers of its allele in descendant nodes.

The method is captured more formally in the following pseudocode:

**Input:** Mixed sample copy number matrix ${\hat{F}}^{m\times \hat{g}}$, tree T (presented as ancestry matrix $A^{n\times n}$ where $A_{i,j}=1$ if node *i* is the ancestor of node *j* and edge matrix $E^{n\times n}$ where $E_{i,j}=1$ if node *i* is the direct parent of node *j*), mapping matrix ${\hat{Q}}^{\hat{g}\times r}$ which maps the unsampled SNVs to segments, frequency matrix $U^{m\times n}$ and component matrix $C^{n\times (l+g+2r)}$ including *g* sampled SNVs.

**Output:** An assignment list $\mathrm{AssignLis}t^{1\times \hat{g}}$, which shows the optimal assignment of nodes for $\hat{g}$ unsampled SNVs (or breakpoints).

**Pseudocode:**
 

$$\begin{array}{c} \hat{C}=C_{:,(l+g+d_{j}r):(l+g+(d_{j}+1)r)}{\hat{Q}}^{T}\\ \overset{ˇ}{C}=C_{:,(l+g+(1-d_{j})r):(l+g+(2-d_{j})r)}{\hat{Q}}^{T}\\ {\hat{C}}^{\text{parent}}=E^{⊤}\hat{C}\\ {\bar{F}}^{i,j,1}=\{\begin{array}{cc} U_{:,i}*{\hat{C}}_{i,j}+UA_{i,:}*{\hat{C}}_{:,j} & \text{if}\;{\hat{C}}_{i,j}=1\;\text{or}\;\\ & {\hat{C}}_{i,j}-{\hat{C}}_{i,j}^{\text{parent}}>1\\ \infty & \text{otherwise} \end{array}\\ {\bar{F}}^{i,j,2}=\{\begin{array}{cc} U_{:,i}*{\hat{C}}_{i,j}+UA_{i,:}*{\hat{C}}_{:,j}/{\hat{C}}_{i,j} & \text{if}\;{\hat{C}}_{i,j}>1\\ \infty & \text{otherwise} \end{array}\\ d_{j}^{\text{opt}},i_{j}^{\text{opt}}={\text{argmin}}_{d_{j}\in \{0,1\},i\in \{1,\ldots ,n\}}\min\{{\bar{F}}^{i,j,1},{\bar{F}}^{i,j,2}\}\\ A\mathrm{ssignLis}t_{j}=i_{j}^{\mathrm{opt}} \end{array}$$

## 3 Results

### 3.1 Validation on simulated data

For validation, we first used simulated data including SVs, SNVs and CNAs based on a pre-defined random evolutionary tree and mutation rate. We mutate whole genome profiles for subclones of a single patient by randomly applying structural variations with lengths sampled from a Poisson distribution with parameter 5 745 000 bp, which is the empirical average length for the TCGA-BRCA cohort with WGS available (Eaton *et al.*, 2018), as well as single-nucleotide variations at uniformly random locations. We simulate allele-specific duplications from 2 to 6, deletions, translocations and inversions with relative probabilities of 2:2:1:1 to ensure a relatively high rate of copy number changes. CNAs occur as consequences of SVs in our model (e.g. from segmental duplications and losses) and are not separately simulated. We also uniformly randomly simulate clonal frequencies for each mixed tumor sample from the same patient. We calculate the theoretical mean copy number for each variant in each sample as a weighted mixture of the copy number of that variant in each sample and its clonal frequency. A theoretical VAF is then calculated for each breakpoint and SNV, as well as a theoretical B-Allele Frequency (BAF) for each CNA. In order to test robustness to noisy sequencing data, we model the read counts *rc* for each segment using a Poisson random variable with parameter of a given read depth 100, then we use a binomial random variable to randomly simulate the read counts that contain a certain copy number alteration, breakpoint or SNV with the probability parameter derived from the calculated theoretical VAF and number of trials of *rc* in the corresponding segment.

We conduct a series of experiments to test robustness of the method to parameter variations, simulating 10 cases for each experiment. We simulate with different structural mutation rates *λ*^total^, which is the parameter of a Poisson distribution for the total number of structural variants. The SNV mutation rate is set to $100\lambda^{\text{total}}$. For each simulation, we generate 1, 5 or 10 samples. We simulate the frequency of each clone for each sample uniformly at random under the frequency constraint and simulate final mixed samples as a weighted sum of the profiles of each clone. We set a maximum run time of 1000 s per iteration and a maximum of 10 iterations, except where specially mentioned below. Each case was run with five random initializations to minimize the risk of solutions being trapped in local optima.

Since there is no other method to our knowledge that integrates all three variant types, direct comparison to alternative methods is challenging. We therefore split validation into two tasks—one assessing performance on CNAs and one on how CNAs contribute to reconstruction from SVs and SNVs—through which we can compare to other work in the literature.

#### 3.1.1 Task 1: validation on segment copy numbers, subclonal frequencies and tree estimation

Here, we assume that the clone number is known and compare our method with CNT-MD (Zaccaria *et al.*, 2018). We also compare with original TUSV to evaluate the improvement specifically from the advances of the present method. Since CNT-MD also uses linear programming for optimization but only utilizes segmental copy numbers, we only compare the estimated copy numbers and clonal frequencies. For fair comparison with CNT-MD, we assumed in our simulations that only leaf nodes and normal clones can have non-zero frequency, setting our method to the leaf-only mode to match the assumptions of CNT-MD.

**Fig. 2.**
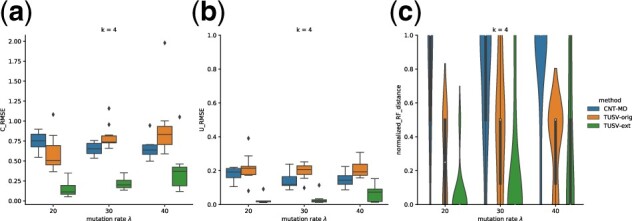
Results on task 1 with varying mutation rates. (**a**) The root mean square error (RMSE) of estimate *C*. (**b**) The root mean square error (RMSE) of estimate *U*. (**c**) Normalized Roubinson-Foulds distance of estimated trees and true tree. All the experiments were run with 1000 s time limit per iteration. Boxes show two quartiles and whiskers show the rest of the distribution except for outliers

We test on different structural mutation rates from 20 to 40 with four leaf clones, and evaluate the performance by the root mean square error of the *U* and *C* matrices including only leaf node profiles and frequencies. The results show that our method can converge faster with the assistance of other variants like SVs and SNVs, showing higher accuracy in estimating both *C* and *U* matrices and phylogenies (Fig. 2). The advantage is larger when the mutation rate is small, which we expect may be more of an issue for CNT-MD because fewer mutations would leave it comparatively less signal from which to deconvolve. Our method also shows superior performance over the original TUSV method, demonstrating the power of taking SNVs and allele-specific variants into account.

**Fig. 3.**
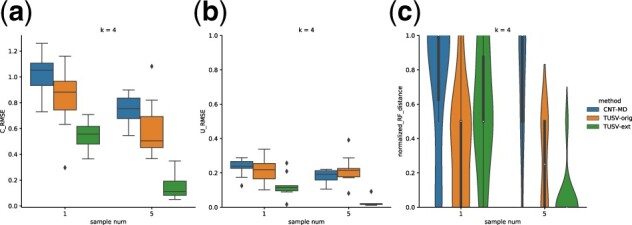
Results on task 1 with varying sample numbers. (**a**) The root mean square error (RMSE) of estimate *C*. (**b**) The root mean square error (RMSE) of estimate *U*. (**c**) Normalized Roubinson-Foulds distance of estimated trees and true tree. All the experiments were run with 1000 s time limit per iteration. Boxes show two quartiles and whiskers show the rest of the distribution except for outliers

All methods have relatively poor performance for sample size 1 compared with sample size 5, suggesting the need for multiple bulk samples for accurate clonal deconvolution. For five samples, all methods improve substantially although TUSV-ext yields notably higher accuracy than CNT-MD and the original TUSV (Fig. 3).

#### 3.1.2 Task 2: validation on SVs, SNVs and numbers of clones

In task 2, we validate the deconvolution of SNVs and SVs as well as determination of number of clones. We sample the SNVs by setting an upper bound on the number of breakpoints and SNVs in total to be 120. We use the average precision of the co-clustering matrix to evaluate the deconvolution performance of both breakpoints and SNVs, as well as the breakpoint-SNVs co-clustering. To determine the correct number of clones, we set the maximum nodes to be 9 when running the program and collapse nodes. We use the relative distance $(k_{\text{true}}-k_{\text{estimate}})/k_{\text{true}}$ as the evaluation metric. We compare our method with PyClone (Roth *et al.*, 2014), which is a popular method for inferring subclonal populations from single or multiple samples, as well as with the original TUSV. We examined the performance among different sample numbers and clone numbers with a fixed mutation rate per branch.

**Fig. 4.**
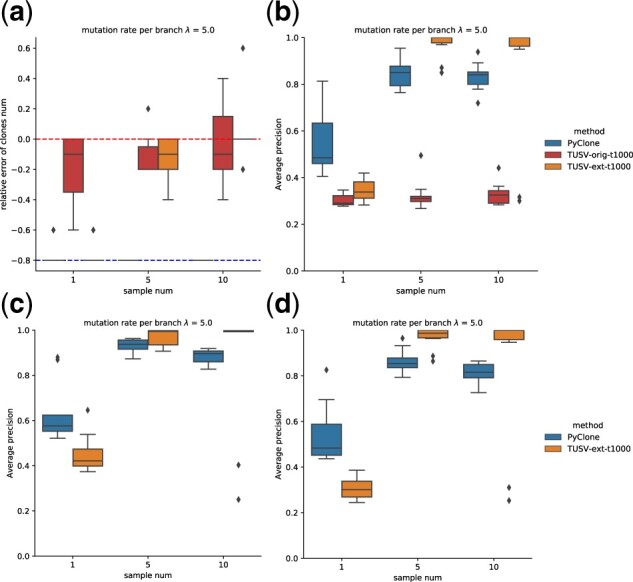
Results on task 2 with different samples size. (**a**) Accuracy of determining number of clones. The upper dashed line shows the best performance of accurately predict the number of clones, and the lower dashed line shows the lower bound of the relative distance when it reaches the maximum clone number. (**b**) Average precision of breakpoints. (**c**) Average precision of SNVs. (**d**) Average precision of breakpoints-SNVs co-clustering. Boxes show two quartiles and whiskers show the rest of the distribution except for outliers

We test on sample sizes 1, 5 and 10 with mutation rate $\lambda^{\text{total}}=30$ and clone number 5. PyClone performs better than TUSV-ext when the sample number is small. When the sample number is 5 or 10, TUSV-ext shows better result in terms of SNVs and breakpoints. PyClone is prone to overestimate the clone number, whereas our method is able to identify the clone number more accurately (Fig. 4). The determination of clone number is also improved when the sample number is larger.

**Fig. 5.**
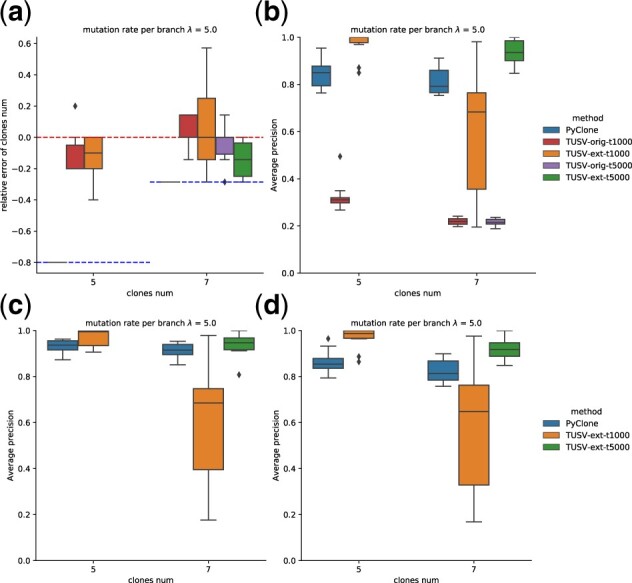
Results on task 2 with different clone numbers (or different total mutation rate) when the mutation rate per branch remains *λ *= 5 (**a**) Accuracy of determining number of clones. The upper dashed line shows the best performance of accurately predict the number of clones, and the lower dashed lines show the lower bound of the relative distance when it reaches the maximum clone number. (**b**) Average precision of breakpoints. (**c**) Average precision of SNVs. (**d**) Average precision of breakpoints-SNVs co-clustering. Boxes show two quartiles and whiskers show the rest of the distribution except for outliers

We then fix the mutation rate per branch to be *λ *= 5 and sample number to be 5 and test for different clone numbers of 5 and 7. For larger clone numbers, resulting in larger number of total variants including breakpoints, we found that TUSV-ext performance notably degraded when using the same setting. We suspected that this was caused by poor convergence due to the larger number of variants. To test this hypothesis, we increased maximum run time per iteration to 5000 for this case, which resulted in improved performance relative to Pyclone (Fig. 5). This result confirmed that the discrepancy is due to poor convergence and that more iterations may be needed for scenarios yielding larger numbers of variants.

### 3.2 Robustness analysis

In order to explore the robustness of the program, we further examined a simulation instance with 7 clones, $\lambda^{\text{total}}=40$, and sequencing depth 100 with respect to four issues: subsample size, subsample, initialization and number of iterations. We ran the scenario with 5 random subsamples, with the upper bound of breakpoints set to 80 and 120 and with the upper bound of sum of breakpoints and SNVs to be 120 and 180, respectively. We tested three time limits of 500, 1000 and 5000 s per iteration for each subsample. For each subsample and each time per iteration, we ran 5 different random starts for 12 iterations in total and recorded the performance for each start after each iteration (Supplementary Figs S1 and S2).

**Fig. 6.**
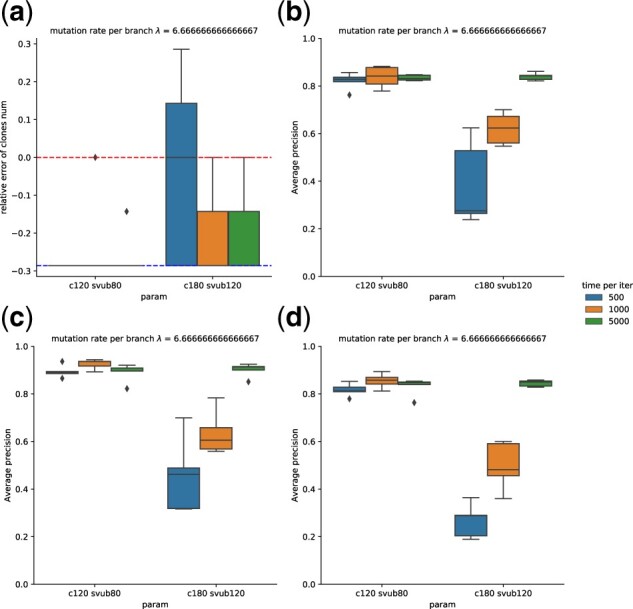
Evaluation of model robustness using simulated data with mutation rate $\lambda^{\text{total}}=40$, 7 clones and 5 random subsamples, with an upper bound on the number of breakpoints of 80 or 120 and an upper bound on the sum of breakpoints and SNVs of 120 or 180, respectively. Time limits of 500, 1000 and 5000 s per iteration were tested for each subsample. Five different random starts were conducted in each setting with 12 iterations. (**a**) Accuracy of determining number of clones. (**b**) Average precision of breakpoints. (**c**) Average precision of SNVs. (**d**) Average precision of breakpoints-SNVs co-clustering. Boxes show two quartiles and whiskers show the rest of the distribution except for outliers

The result shows that in general, different random starts can yield different results (Supplementary Figs S1 and S2). Picking the best of five random starts appears effective in eliminating some trapping of solutions in local minima, as suggested by the low variance of accuracy from different random subsamples (Fig. 6). When the subsampling size is 120 with at most 80 breakpoints, we see no significant difference between different random subsamples (Supplementary Fig. S4), with performance similarly good for each subsample (Fig. 6). When the subsampling size is increased to 180 with at most 120 breakpoints (which should include all the breakpoints), we see a slight improvement in determining the clone number (Fig. 6a). Allowing a longer time limit per iteration seems to boost the performance significantly as well as the tree consistency (Supplementary Fig. S6) when there are larger numbers of variants to accommodate. Yet the best result with more variants seems to be equivalent to the result derived from fewer variants (Fig. 6b–d). It thus appears that one may not need a large number of variants to identify the phylogeny accurately and using more variants may lead to a need for more run time without any improvement in the final result, although we cannot rule out the possibility that increasing run time further could yield improvement with larger numbers of variants.

### 3.3 Application to real prostate cancer data

Gundem *et al.* (2015) performed whole-genome sequencing on 51 tumor samples from 10 patients and provided variant calls of SVs, CNAs, SNVs and Indels. We selected one case from this study, patient A32, to demonstrate our method on real data. For this patient, the study included samples from five tumor regions: (A) right rib metastasis, (C) primary tumor from prostate, (D) left cervical lymph node metastasis, (E) left subclavicular lymph node metastasis and (F) right humerus metastasis.

Since there are many more rearrangements than SNVs, we subsampled both SVs and SNVs for eight runs (approximately 80 breakpoints and 40 SNVs for each run) and mapped the unsampled variants to nodes in the phylogenetic tree. For each run, we set the maximal node count to be 9 and set 10 iterations with time limit of 4000 s per iteration. Each run was performed with two random initializations. We also subsampled copy number segments to retain only those with breakpoints or SNVs lying inside them to reduce the time complexity. We used the two most similar runs, according to the CASet and DISC metrics (DInardo *et al.*, 2020), to identify a consensus solution for subsequent analysis.

**Fig. 7.**
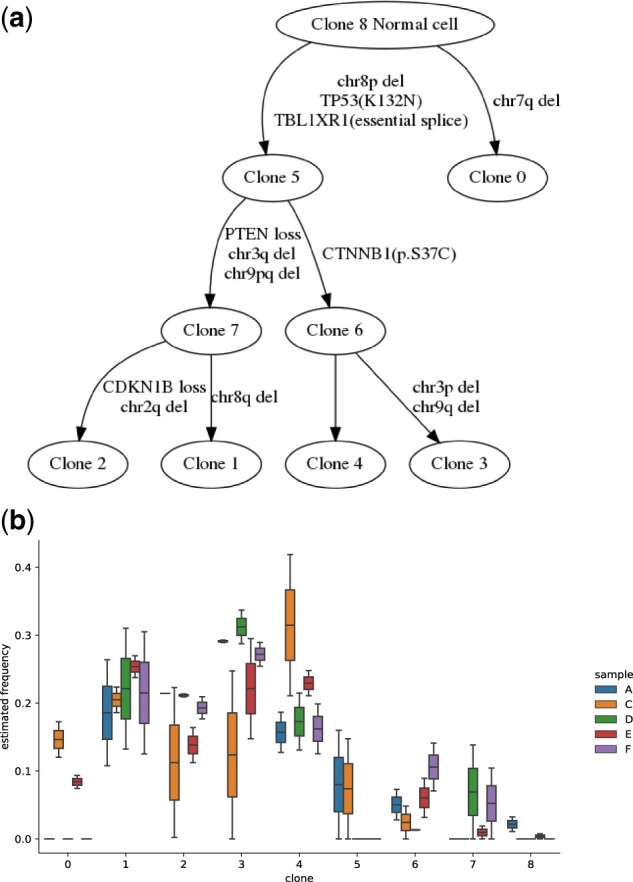
Results for prostate cancer A32 patient data. (**a**) Inferred consensus tree structure with a subset of variants/mutations. (**b**) Comparison of proportions of all 9 clones across samples

We compare the tree inference (Fig. 7a) with that of Gundem *et al.* (2015). Both show branching evolution, yet with distinct trajectories. Clone 0 identified by our method only occurs in samples C and E with relatively low frequencies, while no comparable separate branch is identified in the prior work. Mutations of TP53 (K132N) and TBLIXR (essential splice) and chr8p deletion are inferred as early events in both inferences. Clone 6 was found to contain CTNNB1 substitution. Clones 7 and 2 were inferred to have PTEN and CDKN1B loss respectively, unlike the inferences in the prior work where both PTEN and CDKN1B loss of heterozygosity were inferred to occur early in clonal evolution.

We further examined estimated frequencies of inferred cell populations in each sample (Fig. 7b). The primary tumor (sample C) shows notably higher clonal heterogeneity than the metastases, as we would expect, with evidence for most clones having been present in the primary tumor rather than evolving *de novo* in the metastases. Furthermore, samples A and E are inferred to have small proportions of normal cells (clone 8) while samples C, D and F have no normal cell population. Internal nodes (clone 5, 6 and 7) exhibit low frequencies in all samples. Most leaf nodes (clone 0–5) exhibit higher frequencies in samples C and E and lower in samples A, D and F, or vice versa. Clone 0, the closest child to the diploid normal clone, is not inferred in Gundem *et al.* (2015). Only primary sample C and metastasis E contain this clone and it is expanded in sample C. Clone 4 shows higher proportion in samples C and E than A, D and E, while clones 2 and 3 show the opposite pattern, perhaps suggestive of distinct polyclonal seeding events. Our model overall suggests a more complex pattern of polyclonal origins, whether through polyclonal seeding or subsequent cell migration, than is suggested in the prior work.

## 4 Conclusion

In this work, we develop a tumor phylogeny method, TUSV-ext, to incorporate SNVs, CNAs and SVs into a single clonal lineage tree reconstruction. We show on simulated data how these variant types can be complementary, yielding superior accuracy to competing methods that make use of only subsets of these variants. We further demonstrate effectiveness of the method on a real prostate cancer dataset involving primary and metastatic samples from a single patient. One of the main challenges to the inference is scalability, as the method so far can accommodate relatively limited numbers of variants and potentially requires long convergence time. We offer a heuristic solution for variant subsampling and subsequent mapping, but the method would benefit from more principled solutions for directly handling large variant sets as well as for estimating uncertainty of tree inferences. Furthermore, even with more accurate clonal reconstruction and assignment, some aspects of tumor evolution are not fully resolvable from phylogenetics alone. Incorporating insights from alternative approaches that capture clonal migration (El-Kebir *et al.*, 2018) might be needed to explain data better than is possible by a purely phylogenetic approach such as is presented here.

## Supplementary Material

btac253_Supplementary_Data
