# Supplementary material for: Reconstructing tumor clonal lineage trees incorporating single-nucleotide variants, copy number alterations and structural variations
Source: Bioinformatics. 2022 Jun 27;38(Suppl 1):i125–33. doi: 10.1093/bioinformatics/btac253 (PMC9236577; doi:10.1093/bioinformatics/btac253)
Supplement: btac253_Supplementary_Data [file btac253_supplementary_data.pdf]

Supplementary Methods

S1 Algorithm: Coordinate descent

In this supplement, we elaborate on the ILP described in the main paper, specifically to include components of the program omitted for space from the main text because they are substantially similar to analogous components in the original TUSV Eaton *et al.* (2018). As noted in the main text, we solve for matrices U and C given input matrices F, Q and G by coordinate descent, as in Eaton *et al.* (2018). We first initialize the U matrix randomly under the constraint that $\sum_{k=1}^{N} u_{p,k} = 1, \forall p \in \{1 \ldots m\}$ and solve for C with fixed U. We then fix C and solve for U. The method cycles between these steps iteratively until convergence or until a maximum number of iterations are reached.

S1.1 Algorithm: Estimating clonal frequency matrix U

As described in Eaton *et al.* (2018), we construct an ILP by introducing a set of auxiliary variables $f_{\delta,p,v}$, which describe the element-wise absolute distance between F and UC. U can be solved by minimizing only the first term of the objective function $|F - UC|$, represented by the sum of $f_{\delta,p,v}$, under the constraint that the sum of the frequencies of all clones in each sample is one.

$$f_{\delta,p,v} \geq f_{p,v} - \sum_{k=1}^{N} u_{p,k} c_{k,v}, \forall p \in \{1, \ldots m\}, v \in \{1, \ldots, l+g+2r\} \tag{27}$$

$$f_{\delta,p,v} \geq -f_{p,v} + \sum_{k=1}^{N} u_{p,k} c_{k,v}, \forall p \in \{1, \ldots m\}, v \in \{1, \ldots, l+g+2r\} \tag{28}$$

$$|F - UC| = \sum_{p=1}^{m} \sum_{v=1}^{l+g+2r} f_{\delta,p,v} \tag{29}$$

$$\sum_{k=1}^{N} u_{p,k} = 1, \forall p \in \{1 \ldots m\} \tag{30}$$

S1.2 Algorithm: Estimating copy number matrix C

We then fix U and solve for C given the input matrices F, G and Q.

Phylogenetic constraints: As described in Eaton *et al.* (2018), in order to relate the copy number profiles to the phylogenetic tree T, we define an edge matrix $E^{N \times N}$ describing parent-child relationships in T, where $e_{i,j} = 1$ indicates clone i is the direct parent of clone j. We assume the phylogenetic tree T is a binary tree without loss of generosity.

The root node has no incoming edge (Eq.31) while all other nodes have exactly one incoming edge (Eq.32). Leaf nodes have no outgoing edge (Eq.33) while all other nodes have exactly two outgoing edges (Eq.34). We define the node $N = 2n - 1$ as the root node, nodes 1 to n as leaf nodes, and node $n+1$ to $N-1$ as non-root internal nodes. We can then formulate constraints as follows:

$$e_{i,N} = 0, \forall i \in \{1, \ldots, N\} \tag{31}$$

$$\sum_{i=1}^{N} e_{i,j} = 1, \forall j \in \{1, \ldots, N-1\} \tag{32}$$

$$e_{i,j} = 0, \forall i \in \{1, \ldots, n\}, j \in \{1, \ldots, N\} \tag{33}$$

$$\sum_{j=1}^{N} e_{i,j} = 2, \forall i \in \{n+1, \ldots, N\} \tag{34}$$

We define an ancestor matrix to describe ancestor-descendent relationships in the tree, where $a_{i,j} = 1$ indicates clone i is an ancestor of clone j. We require that the root node has no ancestor and is an ancestor for all other nodes, and that leaf nodes have no descendants (Eq. 35-36). We also require the ancestor matrix to be consistent with the edge matrix. For instance, any parent should be its child's ancestor (Eq. 37) and any ancestor of a parent should be inherited by its children (Eq. 38-39). Those constraints are added to the ILP as follows

$$a_{N,j} = 1, \forall j \in \{1, \ldots, N-1\} \tag{35}$$

$$a_{i,N} = 0, \forall i \in \{1, \ldots, N\} \tag{36}$$

$$a_{i,j} \geq e_{i,j}, \forall i, j \in \{1, \ldots, N\} \tag{37}$$

$$a_{h,j} \geq e_{i,j} + a_{j,i} - 1, \forall i, j \in \{1, \ldots, N\}, g \in \{1, \ldots, i-1, i+1, \ldots, N\} \tag{38}$$

$$a_{h,j} \geq 1 - e_{i,j} + a_{j,i}, \forall i, j \in \{1, \ldots, N\}, g \in \{1, \ldots, i-1, i+1, \ldots, N\} \tag{39}$$

Beyond the common constraints used in Eaton *et al.* (2018), we added two new constraints in the present work to ensure that there is no cycle in the tree,

$$a_{i,j} + a_{j,i} <= 1, \forall i, j \in \{1, \ldots, N\} \tag{40}$$

$$a_{i,i} = 0, \forall i \in \{1, \ldots, N\} \tag{41}$$

Subclone variant copy number constraints: This section is fully presented in the main text and so is not elaborated upon here.

Perfect phylogeny on appearance of breakpoints: We constrain paired breakpoints so that they must occur in the same edge in the tree. We also impose a Dollo phylogeny constraint on SV breakpoints as described in Eaton *et al.* (2018) ensuring that each variant can only appear once although it may be lost multiple times. We treat SNVs as a special case of breakpoints without pairing information and make the same Dollo assumption (Eq. 46). We define $W \in \{0,1\}^{N\times N\times(l+g)}$ to describe the edge on which each breakpoint or SNV occurs, where $w_{i,j,b} = 1$ if breakpoint or SNV b occur at edge from node i to j and 0 otherwise (Eq. 44-45).

We further define a binarization operator as follows

$$\bar{x} = \begin{cases} 1 & x > 0 \\ 0 & x = 0 \end{cases}$$

which can be linearly expressed by an additional variable y_b and a constant x_{max} that upper-bounds the largest possible value of x, where

$$x = \sum_{u=0}^{\lfloor \log_2 x_{max} \rfloor + 1} 2^u y_u \tag{42}$$

$$0 \le y_u \le \bar{x} \le \sum_{v=0}^{\lfloor \log_2 x_{max} \rfloor + 1} y_v, \forall u \in \{0.\ldots, \lfloor \log_2 x_{max} \rfloor + 1\} \tag{43}$$

as described in Zaccaria *et al.* (2018); Eaton *et al.* (2018).

We define a variable X to help define W where

$$x_{i,j,b} = 2 + \bar{c}_{i,b} - \bar{c}_{j,b} - e_{i,j} \tag{44}$$

$$w_{i,j,b} = 1 - \bar{x}_{i,j,b}, \forall i,j \in \{1,\ldots,N\}, b \in \{1,\ldots,l+g\} \tag{45}$$

$$\sum_{i=1}^{N}\sum_{j=1}^{N} w_{i,j,b} = 1, \forall b \in \{1,\ldots,l+g\} \tag{46}$$

In addition, we add constraint that every loss of breakpoint or SNV should co-occur with a allelic-specific segment copy number loss for the segment in which the breakpoint or SNV lies. Similarly, we also constrain breakpoints or SNVs to be duplicated only if their corresponding genome segment is duplicated. See the same section in the main text for more detail.

Structural variant and segment consistency: This section is covered fully in the main text and so is not elaborated upon here.

Supplementary Figures

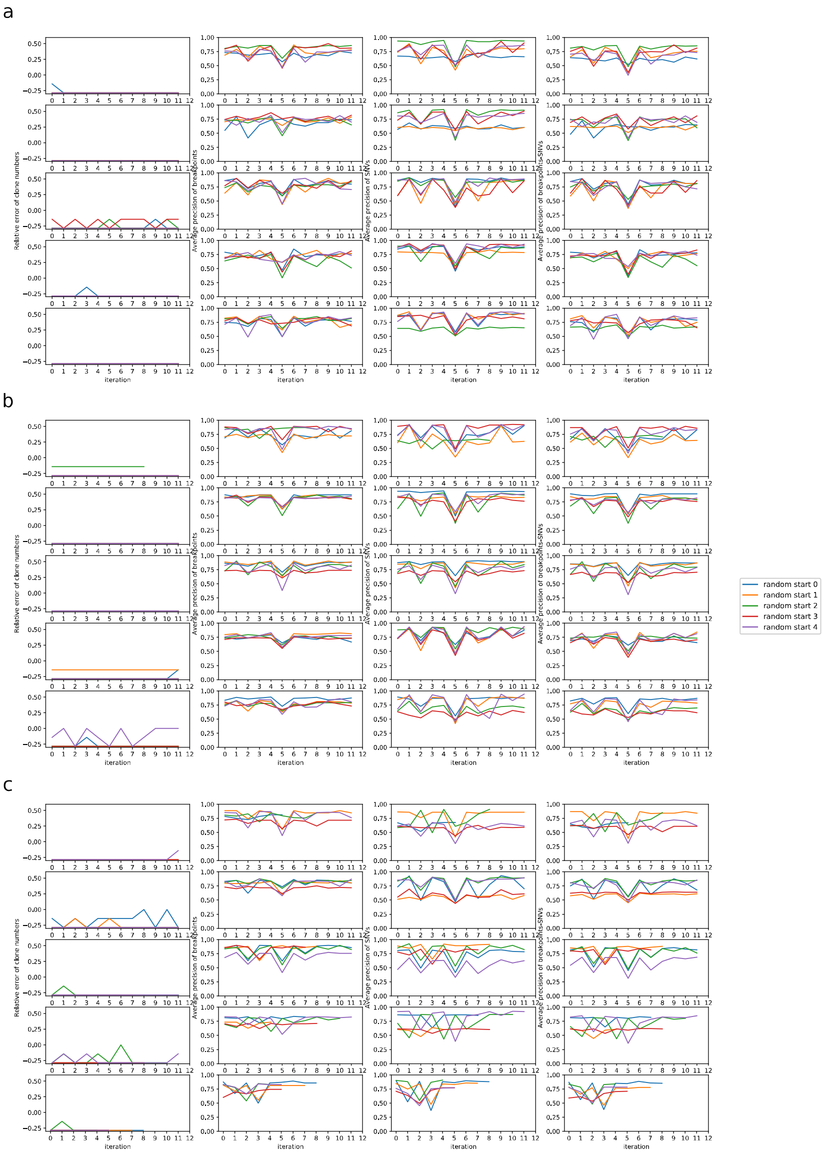

Fig. S1. Performance for different subsamples and different random starts as a function of iteration. Each row represents one random subsample for breakpoints and SNVs and each color represents one random start. The upper bound for breakpoints is set to 80 and the upper bound for breakpoints and SNVs in total is set to 120. The run time limit per iteration is (a) 500, (b) 1000, (c) 5000 seconds per iteration. The evaluation measures are the same as for task2 and each column represents one performance measure.

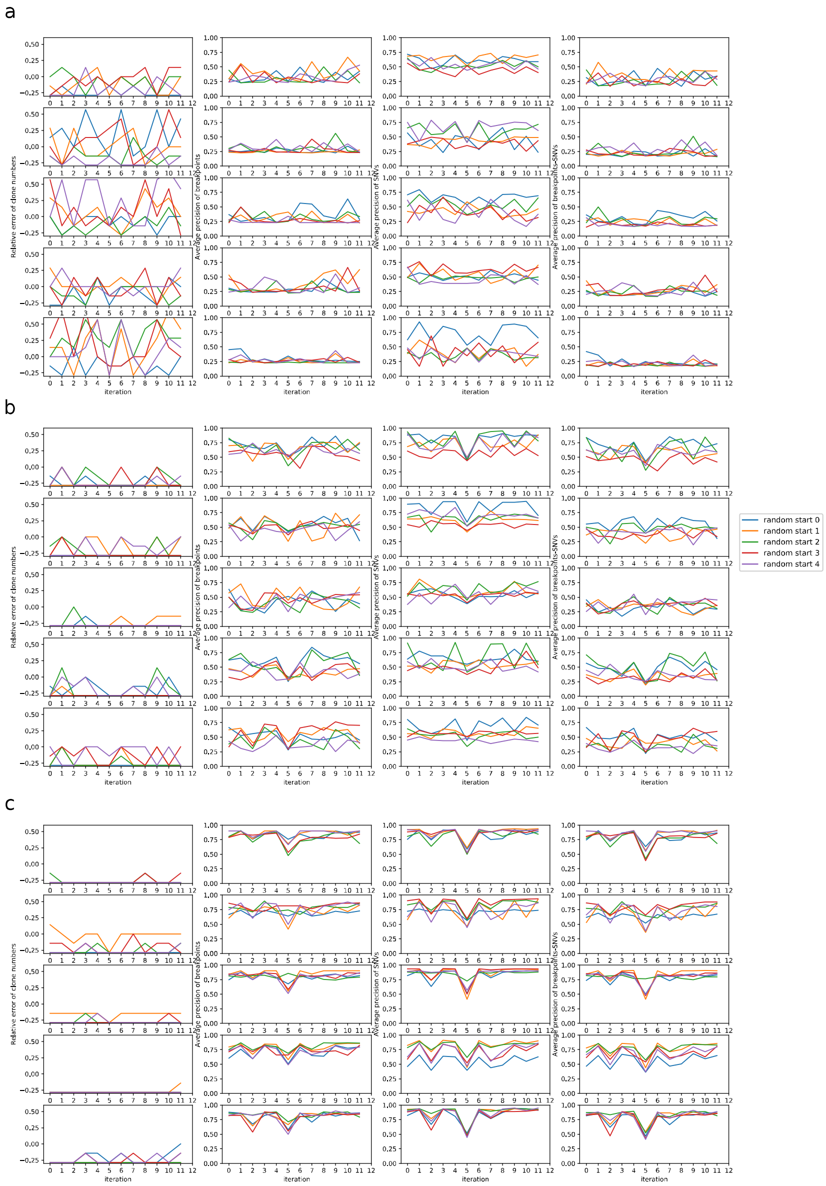

Fig. S2. Performance different different subsamples and different random starts as a function of iteration. Each row represents one random subsample for breakpoints and SNVs and each color represents one random start. The upper bound for breakpoints is set to 120 and the upper bound for breakpoints and SNVs in total is set to 180. Therefore, this example will include all breakpoints for each run but sample different subsets of the SNVs for each subsample. The run time limit is (a) 500, (b) 1000, (c) 5000 seconds per iteration. The evaluation measures are the same as for task2 and each column represents one measure.

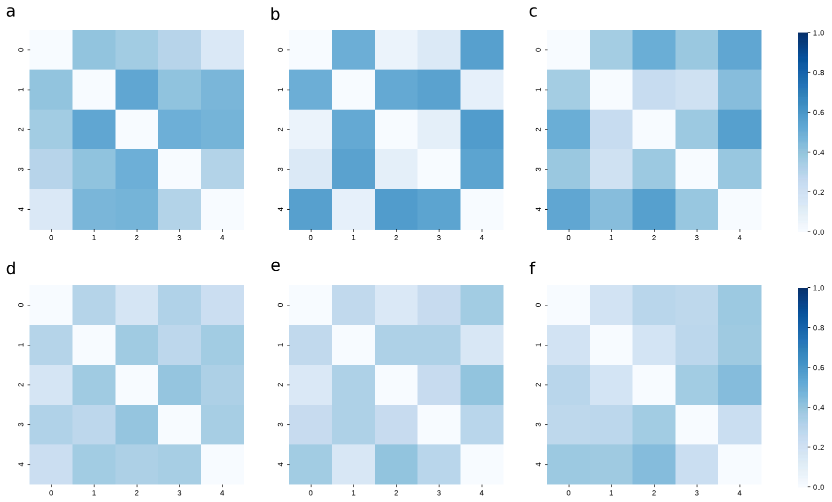

Fig. S3. The inferred tree similarity matrix from different random subsamples evaluated by CASet (a,b,c) and DISC (d,e,f) (DInardo et al., 2020). A larger CASet score represents larger discrepancies between tumor evolutionary trees, emphasizing more on the differences in common ancestors. A larger DISC score also represents larger discrepancies between tumor evolutionary trees, emphasizing more on the differences in more recent mutations. The upper bound for breakpoints is set to 80 and the upper bound for breakpoints and SNVs in total is set to 120. The run time limit is (a, d) 500, (b, e) 1000, (c, f) 5000 seconds per iteration.

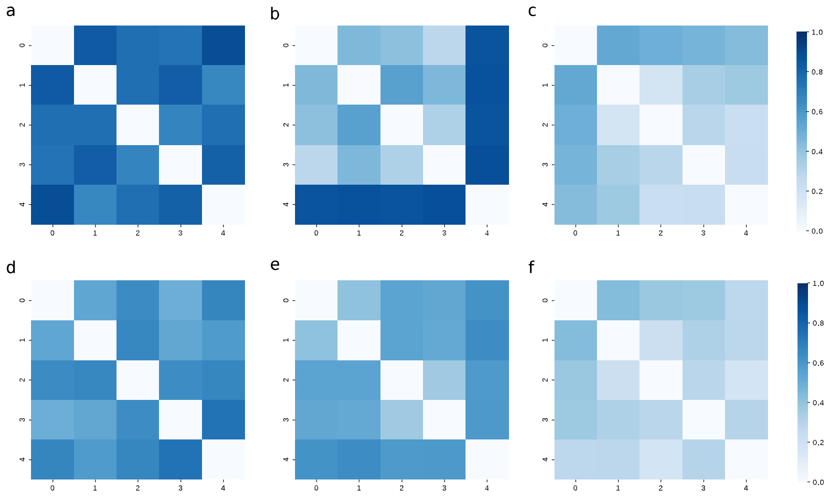

Fig. S4. The inferred tree similarity matrix from different random subsamples evaluated by CASet (a,b,c) and DISC (d,e,f) (DInardo et al., 2020). A larger CASet score represents larger discrepancies between tumor evolutionary trees, emphasizing more on the differences in common ancestors. A larger DISC score also represents larger discrepancies between tumor evolutionary trees, emphasizing more on the differences in more recent mutations. The upper bound for breakpoints is set to 120 and the upper bound for breakpoints and SNVs in total is set to 180. The run time limit is (a, d) 500, (b, e) 1000, (c, f) 5000 seconds per iteration.
